# *Hippophae rhamnoides* L. leaf and twig extracts as rich sources of nutrients and bioactive compounds with antioxidant activity

**DOI:** 10.1038/s41598-022-05104-2

**Published:** 2022-01-20

**Authors:** Małgorzata Kubczak, Ainur B. Khassenova, Bartosz Skalski, Sylwia Michlewska, Marzena Wielanek, Maria Skłodowska, Araylim N. Aralbayeva, Zhanar S. Nabiyeva, Maira K. Murzakhmetova, Maria Zamaraeva, Maria Bryszewska, Maksim Ionov

**Affiliations:** 1 Department of General Biophysics, Faculty of Biology and Environmental Protection, University of Lodz, Lodz, Poland; 2 Department of Biotechnology, Faculty of Food Production, Almaty Technological University, Almaty, Kazakhstan; 3 Department of General Biochemistry, Faculty of Biology and Environmental Protection, University of Lodz, Lodz, Poland; 4 Laboratory of Microscopic Imaging and Specialized Biological Techniques, Faculty of Biology and Environmental Protection, University of Lodz, Lodz, Poland; 5 Department of Plant Physiology and Biochemistry, Faculty of Biology and Environmental Protection, University of Lodz, Lodz, Poland; 6 Faculty of Medicine and Health Care, Farabi Kazakh National University, Al-Farabi av. 71, Almaty, 050040 Kazakhstan; 7 Department of Biophysics and Biomedicine, Faculty of Biology and Biotechnology, Al-Farabi Kazakh National University, Almaty, Kazakhstan; 8 Laboratory of Molecular Biophysics, Department of Microbiology and Biotechnology, Faculty of Biology, University of Bialystok, Bialystok, Poland

**Keywords:** Chemical biology, Plant sciences

## Abstract

Plants have served for centuries as sources of compounds useful for human health such as antioxidant, anti-diabetic and antitumor agents. They are also rich in nutrients that improve the human diet. Growing demands for these compounds make it important to seek new sources for them. *Hippophae rhamnoides* L. is known as a plant with health-promoting properties. In this study we investigated the chemical composition and biological properties of bioactive components of ethanol extracts from leaves and twigs of *H. rhamnoides* L. Chemical components such as the total content of phenolic compounds, vitamins and amino acids and the antioxidant activities of these compounds in cellular and cell-free systems were assessed. The results suggest that the studied extracts are rich in bioactive compounds with potent antioxidant properties. Cytotoxicity and hemotoxicity assays showed that the extracts had low toxicity on human cells over the range of concentrations tested. Interaction with human serum albumin was investigated and conformational changes were observed. Our results indicate that leaf and twig extracts of *H. rhamnoides* L. should be considered as a non-toxic source of bioactive compounds which may be of interest to the food, pharmaceutical and cosmetic industries.

## Introduction

*Hippophae rhamnoides* L., commonly called sea buckthorn, belongs to the *Elaeagnacea*e family^1–3^. It grows mainly in Europe and Asia, preferably in wet places, most often near rivers, as a shrub or low tree^1,4^. This plant is resistant to salinity, drought and air pollution. Oils obtained from both the fruit and leaves of sea buckthorn are rich in chemical compounds that show a wide range of biological activities^1,3–7^. The healing properties of extracts from different organs of sea buckthorn have long been known. In the seventh century BCE, this plant was used not only in natural medicine but also in veterinary treatment^8–11^.

Excess reactive oxygen species (ROS) generation or decreased antioxidant levels in humans cause oxidative stress syndrome, which lead to tissue injury and subsequently lead to pathogenesis of different diseases^12^. Many compounds of plant origin have ROS scavenging properties and could therefore be valuable as protective or therapeutic agents in diseases caused by oxidative stress^13^. Flavonoids and phenolics (phenolic acids) are secondary metabolites formed in many plants that have high antioxidant activity and are known to protect cells specifically against the injurious effects of free radicals^14^. These compounds have been considered to be more effective antioxidants than vitamins C and E or carotenoids^15^. Moreover, flavonoids have been reported not only as powerful antioxidants but also as compounds with multiple biological properties including antimicrobial, cytotoxicity, anti-inflammatory and antitumor activities^16^.

Flavones and catechins are believed to be the most powerful flavonoids for protecting organisms against ROS^13,17^. It has been suggested that, by acting as antioxidants, quercetin (the most abundant dietary flavonol), kaempferol, morin, myricetin, chlorogenic acid and rutin have anti-inflammatory, antiallergic, antiviral and anticancer effects and protect against liver and cardiovascular diseases^18–20^.

Currently, compounds from sea buckthorn are used for prevention or therapy in cardiovascular, skin, liver and stomach diseases^4,21–24^. Extracts from various parts of this plant show great clinical potential because they are rich in compounds with anti-inflammatory, immunomodulatory, radioprotective and anti-tumor activities^1,25–27^. Recently, it has also been shown that sea buckthorn stem extracts have anti-coagulant potential and anti-viral activity^4,5^. Many studies show that compounds in various parts of sea buckthorn have antioxidant activity^8,26,28^. It is now known that the leaves and fruits of sea buckthorn and oils obtained from them are rich in microelements, vitamin A, C and E, lipids, carotenoids, amino acids, unsaturated fatty acids and phenols^4,29^. Moreover, flavonoids, phytosterols, carotenoids and tocopherols can protect organisms against the negative effects of cisplatin and radiation therapy^30–32^, and pectins present in the plant can stimulate the immune system^4^.

However, not all sea buckthorn varieties have such beneficial properties in the same range. The chemical composition can vary significantly in quality and quantity among the plant organs and show variable antioxidant potential^33^.

**Figure 1:**
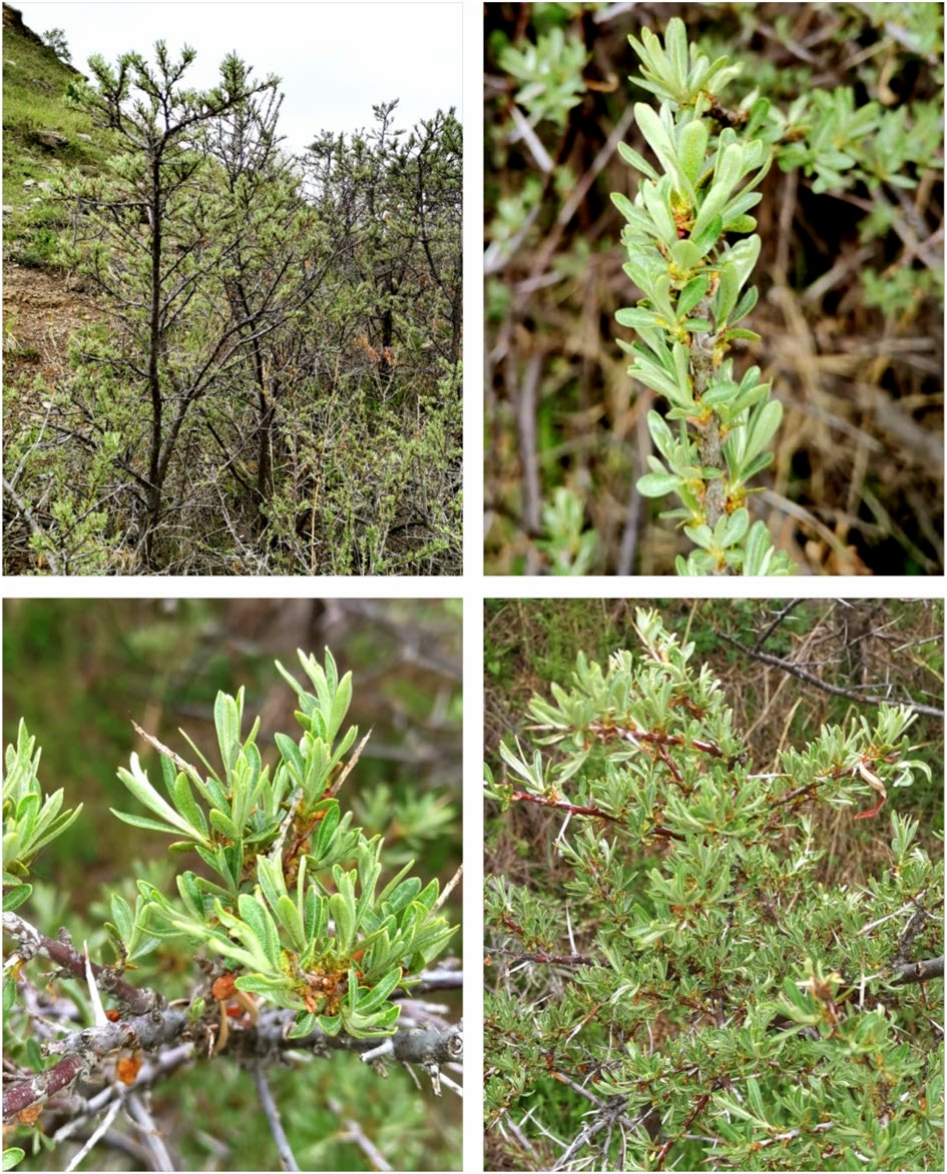
Representative *Hippophae rhamnoides* L. plants and their parts. Location—foothills of the Trans Ili Alatau Mountains (Almaty region, Kazakhstan). The plants were identified and voucher specimen No. 54 (*H. rhamnoides* L.) was deposited at the herbarium of the Institute of Botany and Phytointroduction (Almaty, Kazakhstan).

The present study characterizes the chemical composition and the biological properties of leaf and twig ethanol extracts of *H. rhamnoides* L. plants collected from mountainous areas of the Almaty region, Republic of Kazakhstan in the summer of 2018 (Fig. 1). Their antiradical activities and effects on BJ (human fibroblast cell line) and human erythrocytes were studied.

## Results

### HPLC analysis

HPLC analysis identified 40 phenolic compounds in the extracts of *H. rhamnoides* leaves and twigs, respectively (Table 1). Monomeric and polymeric catechins and gallic acid esters such as epigallocatechin were found in both the leaf and twig extracts.

**Table 1:** Content of phenolic compounds detected using HPLC in the extracts of *H. rhamnoides L.* leaves and twigs.

No. according to HPLC RT order	Phenolic compoud	Synonymus	Quantification wavelengths A 235; 280; 325; 375 nm Em 420 nm (Ex 270 nm)	Content [mg/g] in dry matter of extract	
				Leaf	Twig
1	Gallic acid	(3.4.5-Trihydroxybenzoic acid)	280	1.633 ± 0.149	2.160 ± 0.208
2	*p*-Benzoquinone	(Quinone)	235	2.059 ± 0.189	1.484 ± 0.143
3	*α*-resorcylic acid	(3.5-Dihydroxybenzoic acid)	420	2.302 ± 0.211	12.149 ± 1.191
4	Pyrocatechol	(1.2-Dihydroxybenzene. Catechol)	280	0.248 ± 0.233	0.326 ± 0.041
5	Protocatechuic acid	(3.4-Dihydroxybenzoic acid)	420	0.419 ± 0.041	32.832 ± 3.161
6	Neochlorogenic acid	(*trans*-5-*O*-Caffeoylquinic acid)	325	0.182 ± 0.021	0.830 ± 0.087
7	(−)-epigallocatechin	(monomeric flavan-3-ol)	235	6.035 ± 0.592	6.765 ± 0.651
8	(+)-Catechin	(flavan-3-ol) (monomeric flavan-3-ol)	235	2.052 ± 0.186	14.962 ± 1.444
9	4-hydroxybenzoic acid	–	235	3.923 ± 0.228	1.948 ± 0.198
10	Procyanidin B2	(pentahydroxyflavane); (*cis.cis″-*4.8″-Bi(3.3′.4′.5.7-pentahydroxyflavane) (Polymeric flavan-3-ol)	280	13.933 ± 1.276	15.549 ± 1.498
11	Gentisic acid	(2.5-Dihydroxybenzoic acid)	325	1.380 ± 0.162	0.334 ± 0.006
12	4-Hydroxybenzaldehyde	–	280	0.171 ± 0.016	0.185 ± 0.0187
13	Chlorogenic acid	(*trans*-3-*O*-Caffeoylquinic acid)	325	0.144 ± 0.013	0.058 ± 0.006
14	Vanilic acid	(4-Hydroxy-3-methoxybenzoic acid)	420	0.142 ± 0.015	0.328 ± 0.031
15	*β*-resorcylic acid	(2.4-Dihydroxybenzoic acid)	420	0.278 ± 0.025	0.889 ± 0.091
16	Caffeic acid	(*trans*-3.4-Dihydroxycinnamic acid)	325	0.132 ± 0.014	0.045 ± 0.005
17	(−)-epicatechin	(monomeric flavan-3-ol) ((−)-*cis*-3.3′.4′.5.7-Pentahydroxyflavane)	235	2.353 ± 0.225	1.440 ± 0.123
18	Syringic acid	(4-Hydroxy-3.5-dimethoxybenzoic acid)	420	0.758 ± 0.066	0.902 ± 0.047
19	1.3-Dicaffeoylquinic acid		325	0.182 ± 0.021	0.062 ± 0.006
20	Cyanidin	**(**3.3′.4.5.7-Pentahydroxyflavone) (3.3′.4.5.7-Pentahydroxyflavylium chloride)	280	1.665 ± 0.153	0.974 ± 0.098
21	Syringaldehyde	(4-Hyroksy-3.5-dimethoxybenzaldehyde)	280	0.228 ± 0.024	0.969 ± 0.102
22	*p*-Coumaric acid	(*trans*-4-Hydroxycinnamic acid)	325	0.677 ± 0.069	0.488 ± 0.047
23	Ferulic acid	(4-Hydroxy-3-methoxy-cinnamic acid)	420	0.137 ± 0.014	0.286 ± 0.006
24	Coumarin	(1.2-Benzopyrone)	280	0.311 ± 0.029	0.237 ± 0.023
25	Sinapic acid	(4-Hydroxy-3.5-dimethoxy-cinnamic acid)	420	0.129 ± 0.004	0.552 ± 0.055
26	*trans*-3-Hydroxycinnamic acid	(*m*-Coumaric acid)	280	0.491 ± 0.051	0.122 ± 0.011
27	Luteolin	7-*O-β-d*-glucoside	325	2.018 ± 0.089	0.583 ± 0.061
28	Rutin	(quercetin-3-*O*-rutinoside)	375	7.791 ± 0.731	0.649 ± 0.051
29	Ellagic acid	(4.4′.5.5′.6.6′-Hexahydroxydiphenic acid 2.6.2′.6′-dilactone)	235	4.444 ± 0.416	0.456 ± 0.047
30	Hesperidin	(Hesperetin-7-rutinoside)	280	4.949 ± 0.311	0.610 ± 0.062
32	*o*-Coumaric acid	(*trans*-2-Hydroxycinnamic acid)	420	0.289 ± 0.029	0
31	Rosmarinic acid	(R,E)-3-(3,4-dihydroxyphenyl)-2-((3-(3,4-dihydroxyphenyl)acryloyl)oxy)propanoic acid	420	11.642 ± 1.065	1.452 ± 0.014
33	Salicylic acid	(2-Hydroxybenzoic acid)	420	0.701 ± 0.067	0.961 ± 0.098
34	Myricetin	(flavonol) (3.3′.4′.5.5′.7-Hexahydroxyflavone)	375	0.720 ± 0.075	0.413 ± 0.042
35	Quercetin	(flavonol) (3.3′.4′.5.7-Pentahydroxyflavone)	375	0.149 ± 0.011	0.049 ± 0.005
36	*trans*-Cinnamic acid		280	0.136 ± 0.014	0.094 ± 0.009
37	Naringenin	(4′.5.7-Trihydroxyflavanone)	280	0.019 ± 0.002	0.236 ± 0.032
38	Luteolin	(3′.4′.5.7-Tetrahydroxyflavone)	325	2.772 ± 0.266	0.787 ± 0.077
39	Kaempferol	(3.4′.5.7-Tetrahydroxyflavone)	375	0.193 ± 0.015	0.026 ± 0.003
40	3-Hydroxyflavone	Flavonol	325	0.131 ± 0.016	0.125 ± 0.012

Values are means ± SD (n = 3).

There was a high content of dihydroxybenzoic acids (*α*-resorcylic and protocatechuic acid) in the twig extracts, whereas the leaf extracts had higher contents of rosmarinic (8-fold), genistic (4-fold), chlorogenic (2.5-fold) and ellagic (12-fold) acids than twig extratcs. The amounts of flavonoids such as rutin, luteolin and hesperidin were higher in the leaf than the twig extracts by about 12-fold, 3.5-fold and 8-fold, respectively. In general, the twig extracts showed lower levels of phenolic compounds than in the leaf extracts except for gallic acid and catechin (1.3-fold and 7-fold higher in twigs than leaves, respectively).

### Content of phenolic compounds

**Figure 2:**
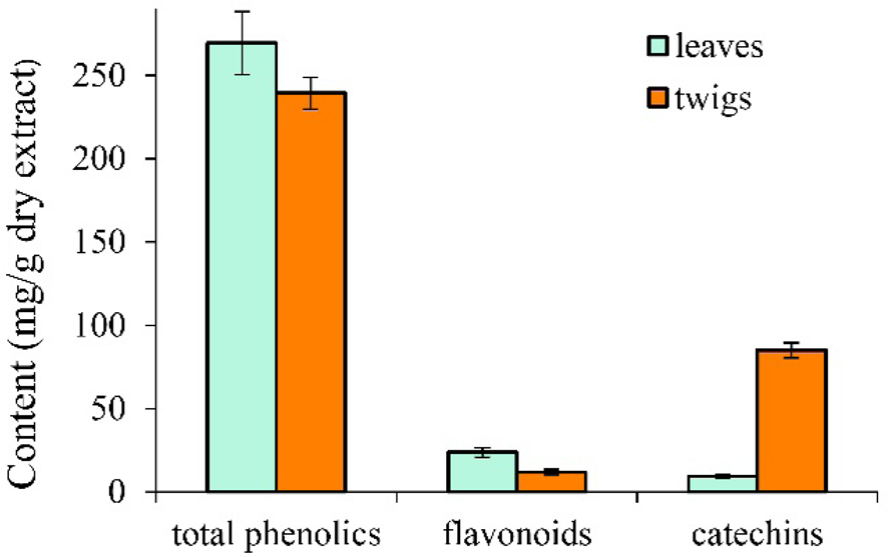
Content of phenolic compounds in the extracts of *Hippophae rhamnoides* L*.* leaves and twigs. Values are means ± SD (n = 3).

The results indicate similar contents of total phenolic compounds in the *H. rhamnoides* leaf and twig extracts (Fig. 2): 269.49 mg/g dry matter for leaf extract and 239.41 mg/g dry matter for twig extract.

The levels of flavonoids were higher in leaf than in twig extracts (23.7 mg/g and 11.8 mg/g for dry matter of extracts, respectively). Flavonoids accounted for about 9% and 5% of the phenolic compounds in the extracts of leaves and twigs, respectively. However, because AlCl_3_ reacts mainly with flavones, flavonols, flavanones and flavanols, the results obtained do not correspond exactly to the total flavonoid contents of the tested extracts.

In contrast to the flavonoids, the catechin concentration was higher in the twig than the leaf extracts (9.3 mg/g and 85 mg/g dry matter of extracts, respectively). The total content of catechins (flavan-3-ols) in the twig extracts was over nine times higher than in the leaf extracts.

### Determination of amino acid content

Table 2 shows the total content of amino acids in leaves and twigs from *H. rhamnoides*. More amino acids were found in leaves (0.62%) than twigs (0.45%). Among all the amino acids, arginine, histidine and proline were most abundant in the twigs (0.087%, 0.058% and 0.067%, respectively), while valine, proline, phenylalanine and arginine were most abundant in the leaves (0.084%, 0.081%, 0.070% and 0.066%, respectively). Due to the method limitation, the content of glutamic and aspartic acid was difficult to evaluate.

**Table 2:** Total amino acid content in dry matter of extracts of twigs and leaves from *Hippophae rhamnoides* L.

Amino acids	Content (mg/g) in dry matter of extract	
	Leaf	Twig
Arginine	6.56 ± 0.08	8.72 ± 0.06
Lysine	5.2 ± 0.07	3.13 ± 0.01
Tyrosine	–	1.00 ± 0.01
Phenylalanine	7.01 ± 0.05	2.90 ± 0.02
Histidine	–	5.81 ± 0.03
Leucine + isoleucine	6.56 ± 0.07	0.12 ± 0.01
Methionine	0.50 ± 0.01	3.35 ± 0.04
Valine	8.37 ± 0 .07	3.13 ± 0.03
Proline	8.14 ± 0.06	6.71 ± 0.05
Threonine	4.52 ± 0.03	2.23 ± 0.01
Serine	4.75 ± 0.04	2.46 ± 0.01
Alanine	4.98 ± 0.02	2.21 ± 0.02
Glycine	5.20 ± 0.03	2.68 ± 0.02
Total content	6.18	4.48

Values are means ± SD (n = 3).

### Determination of water-soluble vitamins and vitamin E isoforms content

The qualitative and quantitative contents of water-soluble vitamins in the leaves and twigs of *H. rhamnoides* are presented in Table 3A. Similar amount of all vitamins analyzed were detected in leaves and twigs. However, the concentrations of vitamin B6 were the highest. Obtained results show that concentration of tocopherol isoforms in the tested plant materials depended of part of plant, moreover, in the case of twig extracts only values for α—tocopherol were detectable (Table 3B). *H. rhamnoides* twigs contained only the α isoform at 1.21 mg/100 g, which was lower than that in the leaves, in which α, ß and γ isoforms were found at 38.2 μg/g, 10.6 μg/g and 8.3 μg/g, respectively.

**Table 3:** Content of water-soluble vitamins (A) and tocopherol isomers (B) in dry matter of extracts of twigs and leaves from *Hippophae rhamnoides* L.

	Water soluble vitamins	
Vitamins	Content (mg/g) in dry matter of extract	
	Leaf	Twig
**(A)**		
B1 (thiamine)	3.0 ± 0.19	0.39 ± 0.06
B2 (riboflavin)	4.0 ± 0.21	0.33 ± 0.05
B3 (niacin)	3.0 ± 0.14	3.8 ± 0.29
B5 (pantothenic acid)	2.8 ± 0.09	2.2 ± 0.08
B6 (pyridoxine)	5.4 ± 0.22	7.6 ± 0.32
Folic acid	0.8 ± 0.07	0.9 ± 0.05

	Vitamin E isomers	
	Isomer	Content, [μg/g] in dry matter of extract
**(B)**		
Leaf	α—tocopherol	38.2 ± 0.22
	ß—tocopherol	10.6 ± 0.18
	γ—tocopherol	8.30 ± 0.05
Twig	α—tocopherol	12.1 ± 0.09
	ß—tocopherol	–
	γ—tocopherol	–

Values are means ± SD (n = 3).

### Antioxidant activity of extracts

**Figure 3:**
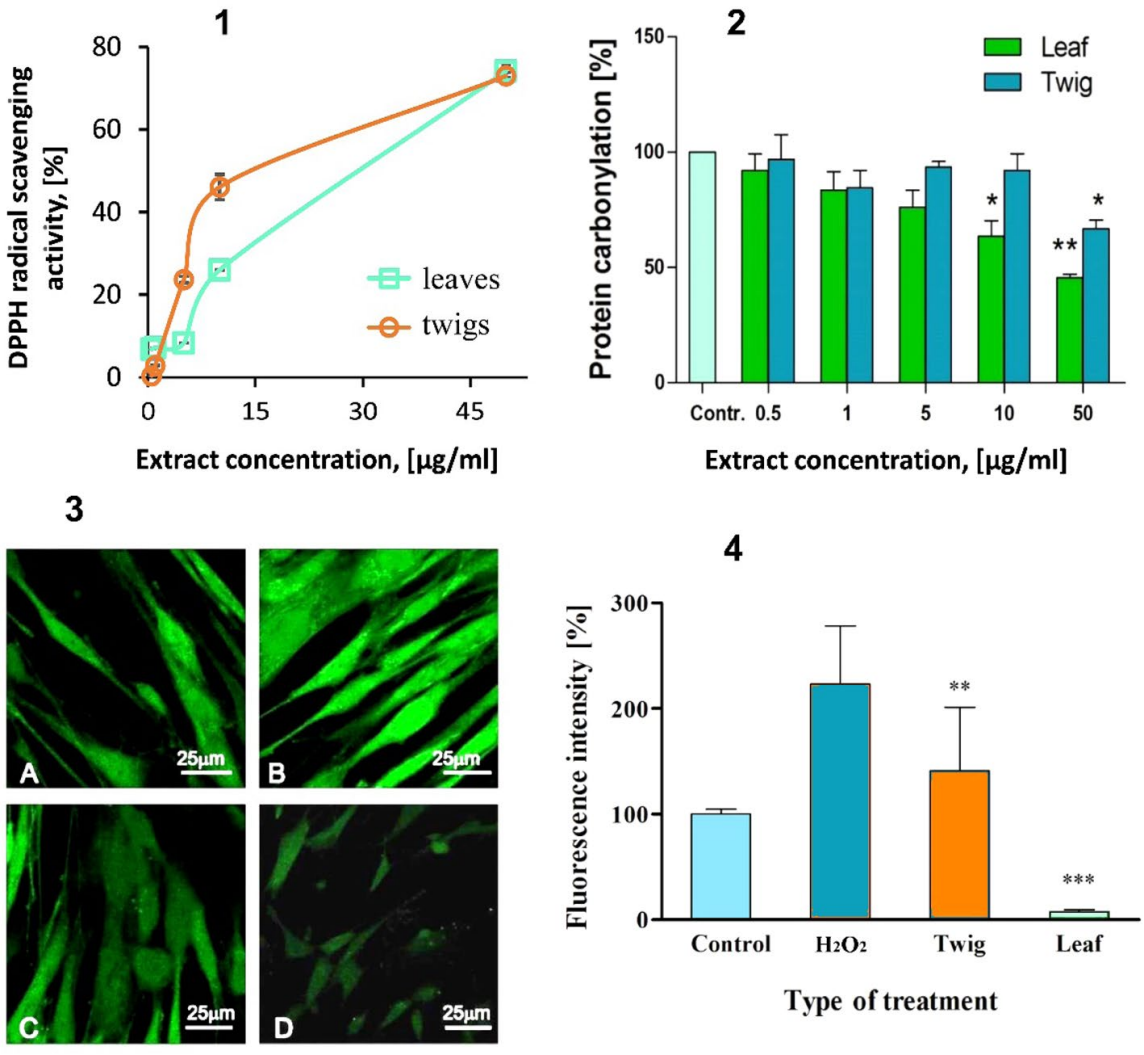
Antioxidant potential of *Hippophae rhamnoides* L. extracts: (1)—Percentage of DPPH free radical scavenging by the leaf and twig extracts of *Hippophae rhamnoides* L. over the concentration range of 0.5–50 µg/ml. Incubation time 10 min. The data represent mean ± SEM of three independent repetitions. (2)—The effect of *Hippophae rhamnoides* L. twig and leaf extracts (0.5–50 μg/ml) on protein carbonylation induced by H_2_O_2_/Fe. The data represent mean ± SEM of three independent repetitions. *p < 0.05. **p < 0.001 versus control. Control—extracts free plasma treated with H_2_O_2_/Fe. (3, 4)—Effect of the *Hippophae rhamnoides* L. twig and leaf extracts on H_2_O_2_/Fe-induced ROS production in BJ cells. Incubation time 24 h. Left panel (3)—Confocal microscopy images of (**A**) control (not treated BJ cells); (**B**) H_2_O_2_, 80 µmol/l; (**C**) Twig extract, 50 µg/ml; (**D**) Leaf extract, 50 µg/ml. Fluorescence intensity of ROS in BJ cells. The data are presented as mean ± SD of 5–7 repeats. **p < 0.001. ***p < 0.0001 versus ROS.

Free radical scavenging activity of the H. rhamnoides leaf and twig extracts (Fig. 3-1) was evaluated using 1,1-diphenyl-2-picryl hydrazyl (DPPH) free radical. The antiradical activity of the extracts was dose-dependent in the range of 0–50 µg/ml and at 50 µg/ml amounted to about 75% for both extracts.

The *H. rhamnoides* leaf and twig extracts affected and significantly decreased H_2_O_2_/Fe-induced plasma protein carbonylation. Twig extract was active in the highest concentration (50 μg/ml). Leaf extract exhibited protective activity when 10 μg/ml was used. (Fig. 3-2).

**Figure 4:**
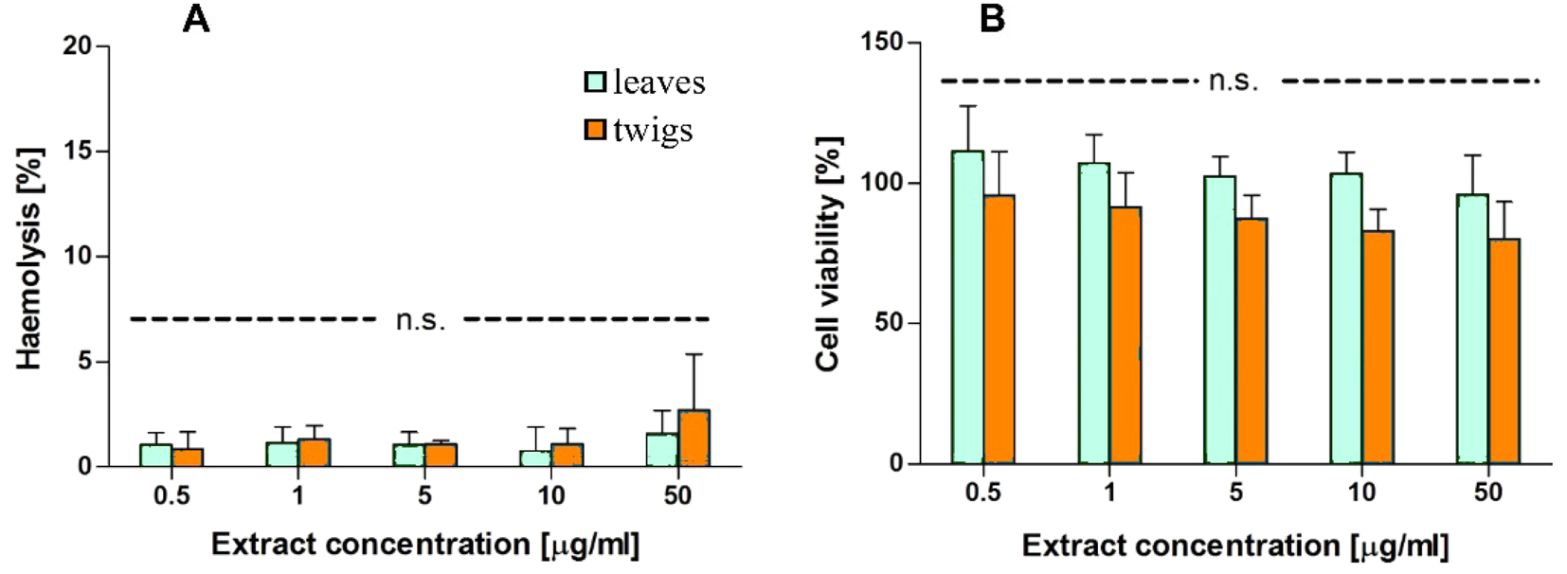
(**A**) The hemolytic activity of the *Hippophae rhamnoides* L. leaf and the twig extracts after 24 h incubation. (**B**) The viability of the BJ cells in the presence of *Hippophae rhamnoides* L. extracts at the concentration ranged from 0.5 to 50 μg/ml. Incubation time 24 h. The data represent mean ± SEM of three independent repetitions. Ns—not statistically significant.

The ability of the H. rhamnoides leaf and twig extracts to decrease ROS production in human fibroblasts was tested with the H_2_DCFDA probe (Fig. 3-3). Both extracts protected the cells against oxidative stress induced by H_2_O_2_. The leaf extract inhibited ROS content in the cells more effectively than twig extract, but both decreased ROS production significantly (Fig. 3-4).

### Hemolysis and cytotoxicity

The extracts of *H. rhamnoides* leaves and twigs were practically non-hemolytic at concentrations ranging from 0.5 to 50 µg/ml (Fig. 4A). The twig extract showed slight hematoxicity at concentration 50 µg/ml, but the level of hemolysis did not exceed 5% after treatment with either extract.

The toxicity of extracts was tested on normal human fibroblast (BJ) cells (Fig. 4B). The *H. rhamnoides* twig extract had a stronger effect on cell viability than the leaf extract but the difference was not statistically significant. The effects did not exceed 20% even after incubation with extracts at the highest concentration (50 µg/ml).

### Interaction with human serum albumin (circular dichroism)

**Figure 5:**
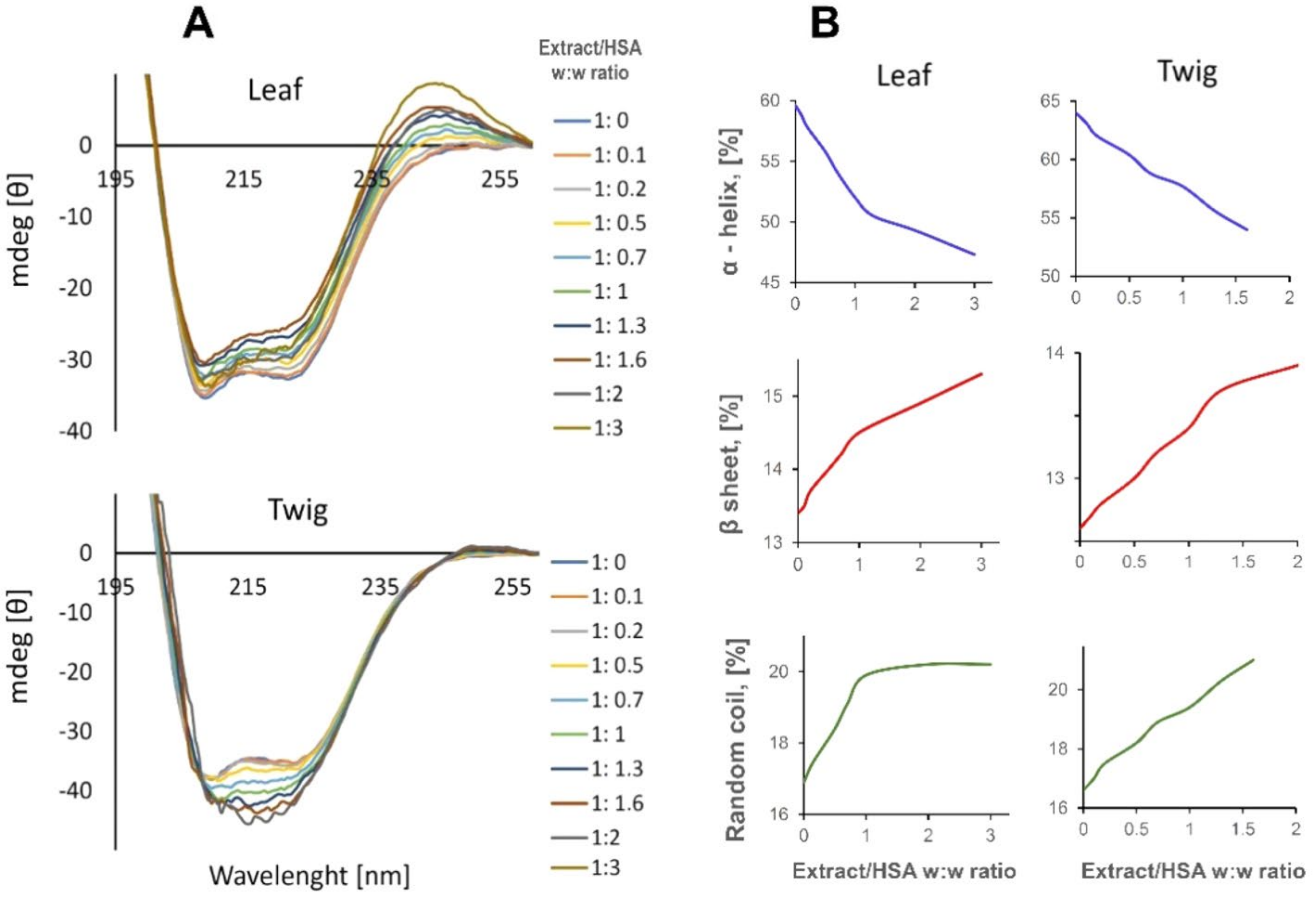
(**A**) Ellipticity changes and (**B**) changes in the secondary structure of HSA at the presence of varying ratios of the *Hippophae rhamnoides* L. leaf and twig extracts. HSA concentration 1 µmol/l.

Human serum albumin exhibits two characteristic negative bands at 202 and 220 nm. Both extracts changed the intensity of these bands that indicated on their interaction with the protein and the effect on its structure. The leaf extract changed the protein secondary structure, (Fig. 5A) more than the twig extract (α-helix content changed from 59.6 to 47.3% and from 64 to 61.4% for the leaf and twig extracts, respectively). The amounts of β-sheet and random coil structures increased from 12.9% to 14.8% and 16.9% to 20.2% respectively for the leaf extract, and from 12.6% and 16.6% to 13% and 17.6%, respectively for the twig extract (Fig. 5B).

## Discussion

The aim of our study was to explore phytochemical content, antioxidant properties and potentially toxic effect on normal human cells of ethanol extracts from *H. rhamnoides* leaves and twigs. *H. rhamnoides* has been known for centuries in folk medicine. It was believed that different parts of this plant have beneficial properties, strongly connected with their chemical composition^28^. The best-known parts of *H. rhamnoides* are the berries and leaves. However, the currently-available literature provides also information about twig extracts from *H. rhamnoides*^5,34,35^.

In order to detect phenolic compounds in leaf and twig extracts from *H. rhamnoides*, HPLC analysis was conducted. The results revealed that the most abundant compounds in the leaf extract were epigallocatechin, procyanidin, epicatechin, luteolin, rutin, ellagic acid and rosmarinic acid. The twig extract showed the highest contents of α-resorcylic acid, protocatechuic acid, epigallocatechin, catechin, procyanidin B2 and epicatechin. According to other studies, the most abundant phenolics in *H. rhamnoides* leaf extract prepared as tea-type infusions are isorhamnetin glucosides and kaempferol^5,33,36,37^. We also detected these compounds, though some of them in lower concentrations. The total phenolic contents of both types of extract were similar but there were differences in the flavonoids and catechins pool. The flavonoid content in the leaf extract was twice that in the twig extract; the total content of catechins (flavan-3-ols) in the twig extracts was over nine times higher than in the leaf extracts. When butanol extracts from *H. rhamnoides* leaves and twigs were investigated, the total phenolics content was higher than we found in ethanol ones (341.5 mg/g and 621.2 mg/g in leaf and twig extracts, respectively)^5,38^. In our study the twig extract contained mainly protocatechuic acid and B-type procyanidin. Similarly, when butanol extracts were tested, catechins and B-type procyanidins were detected in twig extract in the highest concentration^5,38^. Other studies revealed that the butanol extract from leaves was rich in tannins, whereas the butanol extract from twigs was rich in proantocyanidins^5,36^.

Our study showed that rutin and chlorogenic acid are present in both types of extracts but both these compounds are at higher levels in leaves than twigs.

We revealed also three tocopherol isomers (α, β and γ) in the leaf extract, whereas only α-tocopherol was found in the twig extract. In both organs, α isomer was detected in the highest concentration. In another study Kallio et al. described tocopherol contents in berries and seeds, and similarly, Madawala et al. found α-tocopherols in the highest concentration in berry extracts^33,39^. Jaroszewska and Biel estimated the amount of tocopherol in leaves and found 40.98 mg/kg in leaf samples from plants growing in Poland^40^. Vitamins B are a group of water-soluble vitamins that can be found in plant extracts. Plant sources containing vitamins B could be important in the human diet. One of our goals was to measure the total content of vitamins B in leaf and twig extracts from *H. rhamnoides*. Vitamin B6 was the most abundant in both extracts. Vitamins B were detected in low concentrations, so the extracts could serve rather as alternative source of vitamins.

Some parts of plants are discarded as trash, though they could be considered valuable sources of carbohydrates, proteins, fatty acids and other nutrients. Leaves and twigs from most plants are wasted in large amounts by the agro-food industry^41^. We checked the amino acid contents of ethanol extracts from *H. rhamnoides* leaves and twigs. We found eight essential amino acids in twig extracts and seven in leaf extracts. The ratio between essential and total amino acid contents was very similar in both extracts: 63% and 66% in leaf and twig extract, respectively. . Interestingly, the percentage of essential amino acids in both extracts was over 60% in our study. The most abundant essential amino acid in twig extract was arginine (0.8719 g/100 g of dry raw material). In leaf extract, valine (0.8369 g/100 g of dry raw material) was the most representative essential amino acid. Lysine and arginine contents are limited in plant materials, so extracts from different parts of *H. rhamnoides* should be considered attractive sources of amino acids.

It is known that *H. rhamnoides* is particularly interesting owing to its antioxidant potential. Berries, seeds, leaves and twigs could serve as a valuable sources of antioxidant agents^6,42^. Our analysis of the content of bioactive compounds in ethanol extracts of leaves and twigs showed that, in addition to phenolic compounds, they contain several groups of phytochemical that, to varying degrees, exhibit antioxidant properties, namely tocopherols^43^, vitamins^44–46^ and amino acids^47,48^. Tocopherols were found to reduce lipid peroxidation level and to trap the nitric oxide derivatives. Vitamin B6 easily crosses the membranes and is phosphorylated to a coenzyme form. As a coenzyme pyridoxine it is engaged in many chemical reactions of amino acids chains. Ultimately, it is related to the metabolism of cysteine, which is necessary for glutathione formation. The role of glutathione as one of the most potent antioxidant is well established. In the study regarding thiamine the antioxidant capacity was checked. It was demonstrated that vitamin B1 exhibits ·O_2_^−^ and ·OH scavenging activity. Thiamine is also a precursor of cofactor engaged in the oxidative decarboxylation. Product of such reactions—NADPH can directly scavenge radicals and maintain antioxidant power. Folic acid derivatives also exhibited potency to peroxynitrite and inhibit of lipid peroxidation. The role amino acids in antioxidant defense was also studied. The correlation between peptide concentration and improved antioxidant defense was confirmed. It was found that the presence of tyrosine, phenylalanine, histidine, lysine, and arginine correlated with a higher scavenging activity. Moreover, the presence of hydrophobic amino acids and histidine, proline, methionine and cysteine improved antioxidant activity.

Therefore in our study, we checked antiradical scavenging potential of extracts using the DPPH method. Our study revealed that scavenging activity increased with concentration of extracts (0.5–50 μg/ml) and at 50 μg/ml both extracts reduced DPPH to 70%. In Radekov’s study^49^, ethanolic extracts from *H. rhamnoides* leaves/shoots exhibited stronger antiradical activity than extracts from press cake. Shivapriya et al. showed that extracts from leaves/fruits of *H. rhamnoides* exhibited antiradical activity in the concentration range 1–300 μg/ml. The IC_50_ value for antiradical scavenging activity was 70.91 μg/ml^50^.

In order to investigate the antoxidative activity of extracts at cell level , H2DCFDA was used to measure intracellular ROS content induced by hydrogen peroxide in human fibroblasts. The fluorescence level of oxidized H2DCFDA to dichlorofluorescein (DCF) is equal to the amount of ROS generated inside the cells. Extracts used in the highest concentration of 50 μg/ml strongly protected the cells from ROS production. The leaves extract exhibited better protection (*p* < 0.001) than the twig extract (*p* < 0.01) compared with the sample treated with H_2_O_2_ only. In the Shivapriya et al. studies, leaf/fruit extract protected neuronal cells from ROS species however when 100 μg/ml was used and the level of ROS content was decreased by 60–70% (*p* < 0.01)^50^. In our study twig and leaf extract decreased the level of ROS by 62 and 97% respectively comparing to H_2_O_2_-treated cells at 50 μg/ml.

The high level of phenolic compounds in the alcohol extracts of the leaves and twigs of sea buckthorn should provide those extracts with anti-oxidative properties in protecting lipids and proteins. In the present work, the addition of H_2_O_2_/Fe to human plasma leads to increased oxidation of proteins which, in a concentration-dependent manner was inhibited by both extracts. However, the extracts of from leaves inhibited protein carbonylation even in lower concentrations. Phenolic compounds and tocopherols are mainly responsible for the antioxidant activity of the extracts. Their content in the extract from the leaves is higher, therefore, the protective effect of this extract in the case of oxidation of plasma proteins or oxidative stress in fibroblasts is higher than in the case of the extract from the twigs. Early it was shown that butanol extracts from sea buckthorn leaves and twigs inhibited protein carbonylation in vitro^5,38^.

It is known that bioavailability of phenolic compounds is not high. Nevertheless, it has been shown that in vivo application of, for example, flavonoids, they demonstrated a positive effect on human and animal health. And this group of compounds prevents a variety of diseases, including neurodegenerative disorders. The application of phenolic compounds in high concentrations makes possible to achieve micromolar concentrations in plasma. Such concentration is sufficient enough for the realization of their biological effects^51,52^. Absorption of phenolic compounds dependends on many parameters as molecular weight and lipophilicity and stereoisomerization. Low molecular weight compounds as for example gallic acid and lipophilic are more easily absorbed. There are different transport mechanisms of phenolic compounds. They can be absorbed by either passive diffusion or transporters, such as P-glycoprotein and sodium-glucose cotransporters (SGLT), present in the membrane of enterocytes^53^. Both considered extracts from leaves and twigs of *H. rhamnoides* L. contain a large amount of low molecular weight phenolic compounds. Therefore, it can be assumed that these components will be sufficiently absorbed.

Albumins are engaged in transport of both endogenous (fatty acids, hormones) and exogenous compounds in the blood^54^. Checking the interactions between albumins and absorbed chemical compounds is important for understanding and explaining the possibility their transportation and realizing biological activity in the body. In our study we examined the conformational changes in human serum albumin (HSA) upon titration with ethanol extracts using circular dichroism method. We observed slight changes in protein secondary structure: decreased α helix structure and increased β helix and random coil. The results obtained suggested that compounds from the extracts can interact with albumin and cause minor conformational changes. The twig extract interacted little stronger with albumin than the leaves extract. Das et al. also reported that extracts containing flavonoids (quercetin, myricetin, kaempferol) could interact with HSA^54^. This indicates that the plant extracts can easily interact with HSA in the bloodstream without significant altering its structure and transport function and can probably be transported in the body.

Extracts from plants are considered as potential sources of biologically active compounds. Therefore, their safety also should be studied. It is known that plant extracts can be toxic to human cells, so checking their cytotoxicity is very important. Guo et al.^55^ demonstrated that extracts from *H. rhamnoides* berries can decrease cell viability in the HepG2 cell line. However Shivapriya et al.^50^ revealed a neuroprotective effect *H. rhamoides* extract when neuronal cells were treated with hydrogen peroxide and the level of viable cells increased significantly at 100 μg/ml extract. Another study confirmed that leaf and twig extracts from *H. rhamnoides* are non-toxic to mouse fibroblasts^56^. Our study performed on nuclear and non-nuclear cells showed that leaves and twig extracts of *H. rhamnoides* did not change cell viability of fibroblast cell line significantly and do not damage erythrocyte membranes. Therefore, they should be considered as a no-toxic source of beneficial compounds.

## Experimental section

### Plant material and extracts preparations

**Figure 6:**
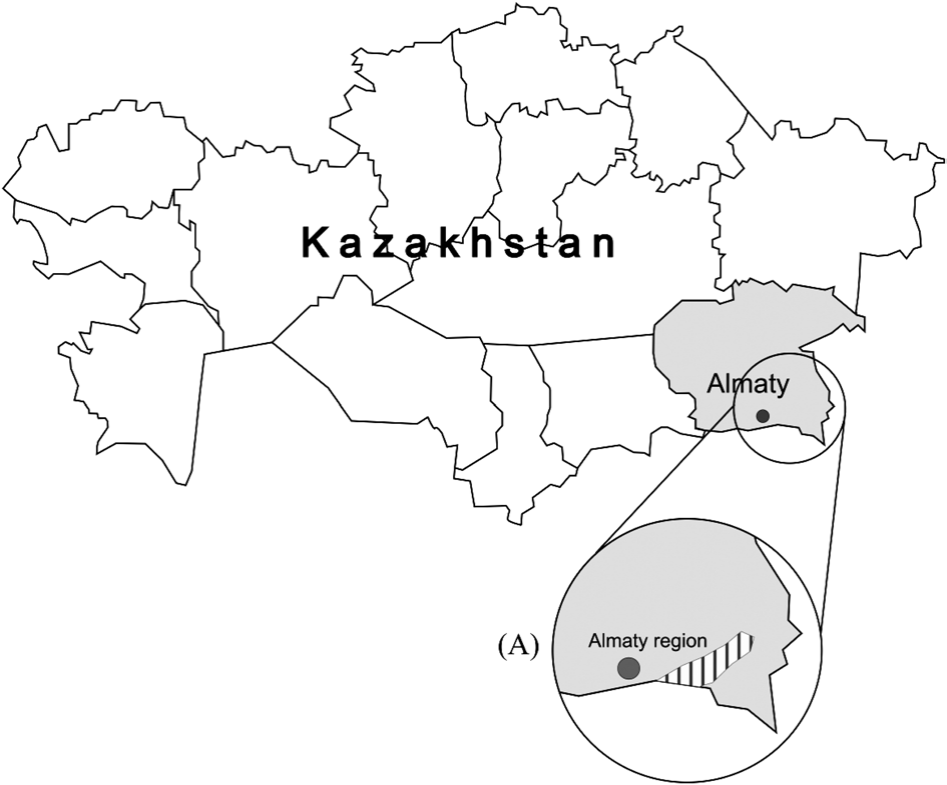
Kazakhstan with the Almaty region (shaded area on the map). A: Study region in southeastern Almaty (lined area on the inset). Map was created using CorelDraw 2017 (www.coreldraw.com/pl/?link=um) licensed by the University of Lodz.

The leaves and twigs of *H. rhamnoides* L. were collected from mountainous areas of Almaty region, Republic of Kazakhstan in the summer of 2018 (Fig. 6) by the Institute of Biology and Biotechnology of Al-Farabi Kazakh National University in accordance with a permission (№ 07-7-1-30-50, from 08.08.2018).

Collection of material from the plant *H. rhamnoides* L., the organization of experimental research and field research for plants (cultural or wild) was carried out in accordance with the Environmental Code of the Republic of Kazakhstan dated January 9, 2007N 212. Aerial parts of *H. rhamnoides* L. were collected in the river Shelek of the Ile Alatau mountain region (Almaty region, Kazakhstan) by Dr. Alibek Ydyrys (based on the permission № 07-7-1-30-50 from Scientific Institute of Biology and Biotechnology). *H. rhamnoides* L. were deposited in voucher specimen No.H-5470,) in the Herbarium of the Research Institute of Biology and biotechnology, Al-Farabi Kazakh National University, Almaty, Kazakhstan. The part of the plant to be analyzed was thoroughly rinsed in distilled water and dried at room temperature under sterile conditions.

The dried, powdered leaves and twigs were extracted with 50% ethanol at room temperature (24 ± 2 °C) on a rotary shaker (110 rpm) for 20 h and centrifuged (20 min/20 000 rpm), then supernatants were dried under vacuum in a rotary evaporator at 50 °C. The dried extracts were stored at 4 °C. An aqueous or ethanol solution was used as a solvent for the extracts, depending on the type of experiment.

### HPLC analysis

HPLC analysis were performed as previously described^57^. The HPLC system (Summit × 2 Dual-Gradient System, Dionex, Sunnyvale, CA, USA) was equipped with a photodiode-array detector (PDA100 DAD) and fluorescence detector (RF-2000). The phenolic compounds present in the extracts were separated on a RP-C18 column (aQ Hypersil GOLD, 250 × 4.6 mm, 5 µm) joined with a guard column (GOLD aQ Drop-In guards, 10 × 4 mm, 5 µm, Polygen, Gliwice, Poland) at 25 °C. The injection volume of the analyzed samples was 20 µl. A mobile phase composed of water (A) and methanol (POCH, Gliwice, Poland) (B), both with 0.1% formic acid (Sigma-Aldrich, Saint Louis, MO, USA) was used. The injection was started after 2 min of isocratic elution with 5% B, increasing slowly over 30 min to 55% B, followed by 5 min of isocratic elution. Between 37 and 47 min the concentration of phase B increased to 70% followed by 5 min isocratic elution. Then between 52 and 54 min the gradient was returned to the initial 5% B and the column was recalibrated for the next 3 min. The flow rate was 1 cm^3^/min. The absorbance was measured at 235, 280, 325 and 375 nm, and the fluorescence at 420 nm (excitation 270 nm). Phenolic compounds in the *H. rhamnoides* extracts were identified by comparing the retention times and on-line UV absorption spectra of the analysed samples with the respective data obtained from reference standards (Sigma-Aldrich, Saint Louis, MO, USA, Fluka, Buchs, Switzerland). For all 40 compounds external calibration was performed. Quantification was based on a calibration curves for standards of phenolic compounds covering the range 5–200 µg cm^-3^; the linearity of the calibration curve was verified by the correlation coefficient (r^2^ ≥ 0.9994). The optimal wavelengths used for the preparation of calibration curve and quantification of individual metabolites are given in Table 1.

### Content of phenolic compounds

The total phenolic content was determined using Folin-Ciocalteu reagent according to Singleton and Rossi^58^. The absorbance of the reaction product was measured at 725 nm and the phenolic content was expressed as milligrams per gram of dried extract based on the calibration curve (r^2^ = 0.9991) prepared for chlorogenic acid (0–200 g cm^-3^) (Sigma-Aldrich, Saint Louis, MO, USA). The results are given as means ± SD (n = 3). The flavonoid content was determined by the aluminium chloride colorimetric method according to Chang et al.^59^. Reagents AlCl_3_ × 6H_2_O and CH_3_COONa were purchased from Chempur, Piekary Śląskie, Poland. The absorbance of the reaction mixture was measured at 415 nm and the flavonoid content was expressed as milligrams per gram of dried extract based on a calibration curve (r^2^ = 0.9995) prepared for quercetin (0–100 g cm^−3^) (Sigma-Aldrich, Saint Louis, MO, USA). The results are given as means ± SD (n = 3). The total catechin (flavan-3-ol) content was determined by the vanillin assay method according to Bakkalbasi et al.^60^. Reagents were purchased from Chempur (Piekary Śląskie, Poland, (methanol, H_2_SO_4_)) and Sigma-Aldrich (Saint Louis, MO, USA, (vanillin)). The absorbance of the reaction mixture was measured at 500 nm and the total flavan-3-ol content was calculated from a calibration curve (r^2^ = 0.9988) prepared using (+)-catechin (0–100 mg cm^-3^) (Sigma-Aldrich, Saint Louis, MO, USA) and expressed as milligrams per gram of dried extract. The results are given as means ± SD (n = 3).

### Determination of amino acid content

The amino acid composition of the leaf and twig extracts of *H. rhamnoides* was determined by capillary electrophoresis method using the “Capel-105/105 M” capillary electrophoresis system (Lumex Analytical Equipment, Russia, St. Petersburg). 100 mg of the extract was hydrolyzed with 10 ml of hydrochloric acid solution (18%) in an oven at 110 °C for 14–16 h, then the samples were cooled and filtered. The hydrolysate was used to obtain phenylisothiocarbamyl derivatives of amino acids. Briefly, 50 ml of the cooled hydrolysate was evaporated to dryness in a stream of warm air. Next 0.15 ml of sodium carbonate solution (0.1 mol/l), 0.3 ml of 1.6% a solution of phenyl isothiocyanate in isopropyl alcohol were added. Next the mixture has been thoroughly mixed until the precipitate dissolves and leave for 35 min at room temperature. Then the solutions were evaporated, dissolved in 0.5 ml of distilled water, centrifuged, and the supernatant was used for analysis. A microvolume of the solution (~ 2 nl) was introduced into the quartz capillary, pre-filled with 30 mM phosphate bufer, 4 mM β-cyclodextrin, pH 7.4 The total length of the capillary was 75 cm, the effective length was 65 cm, and the internal diameter of the capillary 50 μm, the operating voltage applied to the electrodes was 25 kV, detection was by absorbance at 254 nm, under 30 °C. Calibration was performed with a mixture of amino acids from the LAA-21 kit (Sigma-Aldrich, USA). Aminoacid content was calculated using specialized software "Elforan";

### Determination of water-soluble vitamin contents

The vitamin composition of leaf and twig extracts of *H. rhamnoides* was also investigated by Kapel-105 M Lumex. A weighed portion (0.2 g) of the dried extract was extracted with 25 ml a mixture of sodium tetraborate (0.05 M) and sodium sulfite (0.1 M) ( 3:2 V/V) pH 9,2 with stirring. After extraction samples were centrifuged 5 min at 5000–6000 rpm (Eppendorf Mini-Spin) and supernatant was used for analysis. The total length of the capillary was 75 cm, the effective length was 65 cm, and the internal diameter of the capillary 50 μm. Conditions for separation were as follows: buffer, borate pH = 8.9; with 2.3% SDS, temperature + 30 °C, the operating voltage applied to the electrodes was 25 kV. The vitamins were detected by absorbance at 200–240 nm. The device was calibrated using vitamins from Sigma-Aldrich. Vitamin content was calculated using Elforan software.

### Determination of tocopherol isoforms

The total content of vitamin E isoforms in leaf and twig extracts of *H. rhamnoides* was determined by HPLC method using a high-performance Agilent 1200 chromatograph (USA) with a four-channel thermostat pump, fluorometric detector and specialized software. Briefly, 5 g of extracts were subjected to alkaline hydrolysis with 5 ml 50% КOH in the presence 50 ml ethanol 0.25 g vitamin C. The resulting mixture was heated in a water bath under reflux at 80 °C for 40 min. Next, 50 ml of water was added to the analyzed sample and transferred to a separatory funnel. Then the mixture was extracted 3 times with 50 ml of isopropyl alcohol. The combined isopropyl extracts were washed with water four times with a volume of 50 ml. The solvent was removed from the isopropyl extract by distillation using a rotary evaporator. Traces of water were removed by drying with anhydrous sodium sulfate. The dry residue was dissolved in 1 ml of the mobile phase (isopropyl alcohol) and applied to 250 × 4.6 mm Zorbax 300SB-C18 column. The following conditions were selected for determining tocopherol isoforms: flow rate of mobile phase = 0.7 ml/min; column temperature = 35 °C; eluent composition = 42:50:8 acetonitrile: isopropanol: H_2_O. The content of tocopherols was determined using standards from Sigma Aldrich. Detection was carried out at an excitation wavelength of 295 nm and a fluorescence of 330 nm.

### Plasma isolation and determination of carbonyl groups level

Blood samples were obtained from Central Blood Bank, Lodz, Poland. Fresh human blood was collected from healthy volunteers (men and women) not addicted to drugs, tobacco or alcohol. The blood was collected in test tubes containing CPD solution (citrate/phosphate/dextrose; 9:1; v/v blood/CPD) as anticoagulant and centrifuged (1411×*g*, 15 min.). Supernatant from plasma was collected and the protein concentration was calculated from the absorbance at λ = 280 nm according to the Kalckar formula^61^.

The plasma was incubated with the ethanol solution of *H. rhamnoides* leaf and twig extracts at concentrations ranging from 0.5 μg/ml to 50 μg/ml in presence 7.7 mM H_2_O_2_/3.8 mM FeSO_4_/2.5 mM EDTA for 30 min at 37 °C.

Markers of oxidative stress were measured as described previously^57^, briefly:

#### Carbonyl groups measurement

To measure the concentration of carbonyl groups in plasma samples treated with leaf and twig extracts from *H. rhamnoides* and H_2_O_2_/Fe oxidative stress inducers, the colorimetric DNPH method was used; molar absorption coefficient ε = 22,000 M^−1^ cm^−1^. 750 µl of DNPH solution were added to the samples containing plasma, H_2_O_2_/Fe and appropriate volume of the extracts (blank samples were free of DNTP). Next the samples were mixed, incubated in dark for 60 min and centrifuged 5 min at 900x*g* at the presence of 750 µl of 40% TCA. Then the samples were diluted with guanidine and the absorbance was measured. The results are presented as nmol carbonyl group/mg plasma protein. The carbonyl group concentration was measured using the SPECTROstar Nano Microplate Reader, BMG Labtech, Germany.

### Antiradical capacity of extracts measured by reduction of DPPH radical

The antioxidant activity of the extracts was estimated using the modified Brand-Williams method^62^. Synthetic free radical DPPH (2,2′-diphenyl-1-picrylhydrazyl, Sigma Aldrich) was used. The DPPH was dissolved in ethanol to a final concentration 8.3 × 10^–5^ M. The effect of the ethanol solution of extracts from leaves and twigs of *H. rhamnoides* was tested at concentrations in the range 0.5–50 μg/ml after 5, 10, 15, 30 and 45 min. The absorbance was measured at λ = 517 nm. Three independent measurements were done. The level of DPPH reduction was calculated as follows:

$$ \% {\text{ inhibition}}:{1}00 \, \left( {{\text{A}}_{0} {-}{\text{A}}_{{{\text{av}}}} } \right)/{\text{A}}_{0} $$

where A_av_ = the averaged absorbance of samples containing extracts, A_0_ = the absorbance of DPPH solution.

### Haemolysis test

Blood obtained from healthy donors was purchased from the Central Blood Bank, Lodz. It was centrifuged and erythrocytes were washed twice with phosphate buffered saline (PBS), pH 7.4, at 4 °C. Suspension of erythrocytes in PBS with 2% hematocrit was prepared and was used immediately for experiments. *H. rhamnoides* leaf and twig extracts at concentrations in the range 0.5–50 μg/ml were added to the erythrocytes samples and incubated for 24 h at 37 °C. After incubation, hemoglobin release was measured at λ = 540 nm using a BioTek plate reader. Hemolysis was calculated according to the equation:

$$ {\text{H}}\left( \% \right) = \, \left( {{\text{A}}_{{{\text{pb}}}} {54}0{\text{ nm}}/{\text{ A}}_{{{\text{water}}}} {54}0{\text{ nm}}} \right) \times {1}00\% $$

where H(%) is the percentage hemolysis, A_pb_540 nm is the absorbance of the incubated erythrocytes samples, and A_water_540 nm is the absorbance of erythrocytes incubated with distilled water (treated as 100% hemolysis). The data from three independent measurements are presented as mean ± SD.

### Cells

Human fibroblast (BJ) cell line was purchased from ATCC (Manassas, Virginia, USA). The cells were maintained in DMEM F12 (Gibco, Thermo Fisher Scientific, Waltham, MA, USA) in the plastic tissue culture flasks (Falcon, GE Healthcare Life Sciences, Chicago, Illinois, USA) at 37 °C in a humidified atmosphere containing 5%, CO_2_ and 95% air. Both mediums were supplemented with 10% heat-inactivated fetal bovine serum (FBS) (HyClone, GE Healthcare Life Sciences, Chicago, Illinois, USA) contained 1% antibiotic streptomycin-penicillin (Thermo Fisher Scientific, Waltham, MA, USA).

### Reactive oxygen species (ROS) in human fibroblast (BJ) cell lines

BJ cells were seeded in an 8-well LabTek chamber (Thermo Fisher Scientific) at density 2 × 10^5^ and left overnight to adhere. They were then treated with 50 µg/ml *H. rhamnoides* leaf and twig extracts for 24 h. After incubation with the extracts the cells were incubated with 80 µM H_2_O_2_ for 30 min, then washed with PBS (pH 7.4). Subsequently, 5 µM non-fluorescence probe 2′,7-dichlorodihydrofluorescein diacetate (H_2_DCFDA) was added for 20 min in the dark, and after rinsing with PBS intensity of fluorescent dichlorofluorescein (DCF) was examined in a Leica TCS SP8 confocal microscope with a supercontinuum laser at 485 nm excitation and detection emission at 590 nm. Pictures were analysed and quantitate analysis of ROS level (%) was performed using Leica software.

### Cytotoxicity

The cytotoxicity of leaf and twigs extracts from *H. rhamnoides* was estimated using the Alamar Blue assay. The cells were seeded at 10,000 per well and left overnight to adhere. Next, 0.5–50 µg/ml leaf and twig extracts in ethanol were added. After 24 h, incubation at the 37 °C resazurin sodium salt was added to the final concentration 0.0125% and the absorbance was measured at λ = 595 nm.

Viability was calculated as:

$$ \% {\text{ viability}} = \left( {{\text{A}}}/{{\text{A}}}_{{\text{c}}}  \right) \times 100\% $$

where A is the absorbance of the sample and A_c_ is the absorbance of control, untreated cells. Three independent experiments were performed. The results are presented as mean ± SD.

### Circular dichroism

CD spectra of changes in albumin structure were obtained with a Jasco J-815 CD spectrometer. The *H. rhamnoides* leaf and twig extracts were added at increasing concentrations (3.75 to 112.5 µg/ml) to the albumin solution in 10 mmol/l Na-phosphate buffer, pH 7.4. Spectra were obtained between 260 and 195 nm with a 0.5 cm path length Helma quartz cell. The recording parameters were as follows: scan speed, 50 nm/min; step resolution, 0.5 nm; response time, 4 s.; band width, 1 nm, and slit auto^63,64^. The CD spectra were corrected against the baseline with buffer only. Mean residue ellipticity, θ (cm^2^/dmol), was calculated using software provided by Jasco. The θ/θ_0_ ratio was calculated and plotted as a function of albumin/extract mass ratio.

### Statistical analysis

GraphPad Prism 5.0 and Statistica 13.1 were used for statistical analyses. Non-parametric ANOVA (Kruskal–Wallis test) was used to assess the significance of differences. The significance level was *p* < 0.05.

### Compliance with ethical standards

Blood samples were obtained from the Association of Honorary Blood Donors in Lodz, (Central Blood Bank in Lodz, Poland) from 25.09.2019 to 14.01.2020. To conduct current study the samples were obtained randomly. The blood was collected from healthy volunteers and carefully tested before using in the laboratory. All the experiments published in this manuscript comply with the current laws of the country in which they were performed. The study was approved by the Ethics Committee of the University of Lodz, Poland (NR19/KBBN-UŁ/III/2019). All methods were performed in accordance with the relevant guidelines and regulations.

### Ethics approval and consent to participate

All experimental procedures were approved by the Ethics Committee of the University of Lodz, Poland (NR19/KBBN-UŁ/III/2019).

## Conclusion

The aim of the present work was to determine the chemical composition and biological properties of leaf and twig extracts from *H. rhamnoides* L. Both extracts contained similar compositions of phenolics, but in different proportions. Generally, twig extracts contained more catechins than leaf extracts. Essential and non-essential amino acids were found, along with members of the B vitamins family. We also found tocopherol’s isoforms in both extracts. However, leaf extract contained all three isoforms, whereas only α-tocopherol was detected in twig extract. Antioxidant properties of both extracts were confirmed by the DPPH assay, the H_2_DCFDA method and protein carbonylation assay. Circular dichroism revealed that bioactive compounds from leaf and twig extracts interacted with albumin slightly modifying the secondary structure. Additionally, the lack of toxic effect of *H. rhamnoides* L. extracts on human erythrocytes and normal human fibroblast was demonstrated. In view of the results obtained it can be suggested that extracts from *H. rhamnoides* L. could serve as a non-toxic source of beneficial compounds for a food supplement and cosmetics.

## Data Availability

All data generated or analyzed during this study are included in this published article. Raw data are available on request from the authors.

## References

[1] Mishra KP (2011). A comparative analysis of immunomodulatory potential of seabuckthorn leaf extract in young and old mice. Biomed. Aging Pathol..

[2] Krejcarová J, Straková E, Suchý P, Herzig I, Karásková K (2015). Sea buckthorn (*Hippophae rhamnoides* L.) as a potential source of nutraceutics and its therapeutic possibilities—A review. Acta Vet. Brno.

[3] Jayashankar B, Mishra KP, Ganju L, Singh SB (2014). Supercritical extract of Seabuckthorn Leaves (SCE200ET) inhibited endotoxemia by reducing inflammatory cytokines and nitric oxide synthase 2 expression. Int. Immunopharmacol..

[4] Olas B (2016). Sea buckthorn as a source of important bioactive compounds in cardiovascular diseases. Food Chem. Toxicol..

[5] Skalski B (2019). Biological properties of *Elaeagnus rhamnoides* (L.) A. Nelson twig and leaf extracts. BMC Complement. Altern. Med..

[6] Zeb A, Ullah S (2015). Sea buckthorn seed oil protects against the oxidative stress produced by thermally oxidized lipids. Food Chem..

[7] Larmo P (2019). Effects of a sea buckthorn oil spray emulsion on dry eye. Contact Lens Anterior Eye.

[8] Usha T (2014). Molecular docking studies of anti-cancerous candidates in *Hippophae rhamnoides* and *Hippophae salicifolia*. J. Biomed. Res..

[9] Olas B (2017). Inhibition of blood platelet adhesion by phenolics’ rich fraction of *Hippophae rhamnoides* L. fruits. J. Physiol. Pharmacol..

[10] Pirvu L, Panteli M, Rasit I, Grigore A, Bubueanu C (2015). The leaves of *Aronia melanocarpa* L. and *Hippophae rhamnoides* L. as source of active ingredients for biopharmaceutical engineering. Agric. Agric. Sci. Procedia.

[11] Gupta A, Kumar R, Pal K, Banerjee PK, Sawhney RC (2005). A preclinical study of the effects of seabuckthorn (*Hippophae rhamnoides* L.) leaf extract on cutaneous wound healing in albino rats. Int. J. Low. Extrem. Wounds.

[12] Halliwell B (2009). Antioxidants and human disease: A general introduction. Nutr. Rev..

[13] Tungmunnithum D, Thongboonyou A, Pholboon A, Yangsabai A (2018). Flavonoids and other phenolic compounds from medicinal plants for pharmaceutical and medical aspects: An overview. Medicines.

[14] Apak R (2007). Comparative evaluation of various total antioxidant capacity assays applied to phenolic compounds with the CUPRAC assay. Molecules.

[15] Dai J, Mumper RJ (2010). Plant phenolics: Extraction, analysis and their antioxidant and anticancer properties. Molecules.

[16] Kalinowska M, Bielawska A, Lewandowska-Siwkiewicz H, Priebe W (2014). Plant physiology and biochemistry apples: Content of phenolic compounds vs. variety, part of apple and cultivation model, extraction of phenolic compounds, biological properties. Plant Physiol. Biochem..

[17] Rice-Evans CA, Miller NJ, Paganga G (1996). Structure-antioxidant activity relationships of flavonoids and phenolic acids. Free Radic. Biol. Med..

[18] Materska M (2008). Quercetin and its derivatives: Chemical structure and bioactivity—a review. Pol. J. Food Nutr. Sci..

[19] Sharififar F, Dehghn-Nudeh G, Mirtajaldini M (2009). Major flavonoids with antioxidant activity from *Teucrium polium* L. Food Chem..

[20] Panzella L, Petriccione M, Rega P, Scortichini M, Napolitano A (2013). A reappraisal of traditional apple cultivars from Southern Italy as a rich source of phenols with superior antioxidant activity. Food Chem..

[21] Wang X (2018). Seabuckthorn berry polysaccharide extracts protect against acetaminophen induced hepatotoxicity in mice via activating the Nrf-2/HO-1-SOD-2 signaling pathway. Phytomedicine.

[22] Xu X (2007). Effects of sea buckthorn procyanidins on healing of acetic acid-induced lesions in the rat stomach. Asia Pac. J. Clin. Nutr..

[23] Hao XY (2019). Effects of sea buckthorn pomace supplementation on energy partitioning and substrate oxidation in male lambs. Anim. Feed Sci. Technol..

[24] Czaplicki S, Ogrodowska D, Zadernowski R, Konopka I (2017). Effect of Sea-Buckthorn (*Hippophaë rhamnoides* L.) pulp oil consumption on fatty acids and vitamin A and E accumulation in adipose tissue and liver of rats. Plant Foods Hum. Nutr..

[25] Nogala-Kałucka M (2013). Antioxidant synergism and antagonism between tocotrienols, quercetin and rutin in model system. Acta Aliment..

[26] Olas B, Skalski B, Ulanowska K (2018). The anticancer activity of sea buckthorn [*Elaeagnus rhamnoides* (L.) A. Nelson]. Front. Pharmacol..

[27] Kwon EY (2017). Seabuckthorn leaves extract and flavonoid glycosides extract from seabuckthorn leaves ameliorates adiposity, hepatic steatosis, insulin resistance, and inflammation in diet-induced obesity. Nutrients.

[28] Khan BA, Akhtar N, Mahmood T (2010). A comprehensive review of a magic plant, *Hippophae rhamnoides*. Pharmacogn. J..

[29] Górnaś P, Šnē E, Siger A, Segliņa D (2016). Sea buckthorn (*Hippophae rhamnoides* L.) vegetative parts as an unconventional source of lipophilic antioxidants. Saudi J. Biol. Sci..

[30] Aksoy AN (2015). The role of antioxidant activity in the prevention and treatment of infertility caused by cisplatin in rats. Gynecol. Obstet. Invest..

[31] Chawla R (2007). Radioprotective and antioxidant activity of fractionated extracts of berries of *Hippophae rhamnoides*. J. Med. Food.

[32] Saini M (2010). Hippophae leaf extract concentration regulates antioxidant and prooxidant effects on DNA. J. Diet. Suppl..

[33] Madawala SRP (2018). Impact of location on composition of selected phytochemicals in wild sea buckthorn (*Hippophae rhamnoides*). J. Food Compos. Anal..

[34] Sadowska B, Budzyńska A, Stochmal A, Żuchowski J, Różalska B (2017). Novel properties of *Hippophae rhamnoides* L. twig and leaf extracts—Anti-virulence action and synergy with antifungals studied in vitro on *Candida* spp. model. Microb. Pathog..

[35] Skalski B (2019). Anti-platelet properties of phenolic extracts from the leaves and twigs of *Elaeagnus rhamnoides* (L.) A. Nelson. Molecules.

[36] Skalski B (2019). Isorhamnetin and its new derivatives isolated from sea buckthorn berries prevent H_2_O_2_/Fe—Induced oxidative stress and changes in hemostasis. Food Chem. Toxicol..

[37] Ma X (2019). Phenolic compounds and antioxidant activities of tea-type infusions processed from sea buckthorn (*Hippophaë rhamnoides*) leaves. Food Chem..

[38] Skalski B, Kontek B, Olas B, Zuchowski J, Stochmal A (2018). Phenolic fraction and nonpolar fraction from sea buckthorn leaves and twigs: Chemical profile and biological activity. Future Med. Chem..

[39] Kallio H, Yang B, Peippo P, Tahvonen R, Pan R (2002). Tocotrienols in berries and seeds of two subspecies (ssp. sinensis and mongolica) of sea buckthorn (*Hippophae rhamnoides*). J. Agric. Food Chem..

[40] Jaroszewska A, Biel W (2017). Chemical composition and antioxidant activity of leaves of mycorrhized sea-buckthorn (*Hippophae rhamnoides* l.). Chil. J. Agric. Res..

[41] Prandi B (2019). Food wastes from agrifood industry as possible sources of proteins: A detailed molecular view on the composition of the nitrogen fraction, amino acid profile and racemisation degree of 39 food waste streams. Food Chem..

[42] Olas B (2018). Comparative chemical composition, antioxidant and anticoagulant properties of phenolic fraction (a rich in non-acylated and acylated flavonoids and non-polar compounds) and non-polar fraction from *Elaeagnus rhamnoides* (L.) A. Nelson fruits. Food Chem..

[43] Mathur P, Ding Z, Saldeen T (2015). Tocopherols in the prevention and treatment of atherosclerosis and related cardiovascular disease. Clin. Cardiol..

[44] Jung IL, Kim IG (2003). Thiamine protects against paraquat-induced damage: Scavenging activity of reactive oxygen species. Environ. Toxicol. Pharmacol..

[45] Stocker P, Lesgards JF, Vidal N, Chalier F, Prost M (2003). ESR study of a biological assay on whole blood: Antioxidant efficiency of various vitamins. Biochim. Biophys. Acta Gen. Subj..

[46] Rezk BM, Haenen GRMM, Van Der Vijgh WJF, Bast A (2003). Tetrahydrofolate and 5-methyltetrahydrofolate are folates with high antioxidant activity. Identification of the antioxidant pharmacophore. FEBS Lett..

[47] Tejpal CS (2017). Antioxidant, functional properties and amino acid composition of pepsin-derived protein hydrolysates from whole tilapia waste as influenced by pre-processing ice storage. J. Food Sci. Technol..

[48] Wu HC, Chen HM, Shiau CY (2003). Free amino acids and peptides as related to antioxidant properties in protein hydrolysates of mackerel (*Scomber austriasicus*). Food Res. Int..

[49] Radenkovs V, Püssa T, Juhnevica-Radenkova K, Anton D, Seglina D (2018). Phytochemical characterization and antimicrobial evaluation of young leaf/shoot and press cake extracts from *Hippophae rhamnoides* L. Food Biosci..

[50] Shivapriya S, Ilango K, Dubey GP (2015). Evaluation of antioxidant and neuroprotective effect of *Hippophae rhamnoides* (L.) on oxidative stress induced cytotoxicity in human neural cell line IMR32. Saudi J. Biol. Sci..

[51] Manach C, Scalbert A, Morand C, Rémésy C, Jiménez L (2004). Polyphenols: Food sources and bioavailability. Am. J. Clin. Nutr..

[52] Manach C, Williamson G, Morand C, Scalbert A, Rémésy C (2005). Bioavailability and bioefficacy of polyphenols in humans. I. Review of 97 bioavailability studies. Am. J. Clin. Nutr..

[53] Cosme P, Rodríguez AB, Espino J, Garrido M (2020). Plant phenolics: Bioavailability as a key determinant of their potential health-promoting applications. Antioxidants.

[54] Das P, Chaudhari SK, Das A, Kundu S (2018). Interaction of Flavonols with Human Serum Albumin : A biophysical study showing structure activity relationship and enhancement when coated on silver nanoparticles. J. Biomol. Struct. Dyn..

[55] Guo R, Guo X, Li T, Fu X, Liu RH (2017). Comparative assessment of phytochemical profiles, antioxidant and antiproliferative activities of Sea buckthorn (*Hippophaë rhamnoides* L.) berries. Food Chem..

[56] Żuchowski J, Pecio Ł, Marciniak B, Kontek R, Stochmal A (2019). Unusual isovalerylated flavonoids from the fruit of sea buckthorn (*Elaeagnus rhamnoides*) grown in Sokółka, Poland. Phytochemistry.

[57] Kubczak M (2020). Bioactive compounds and antiradical activity of the *Rosa canina* L. leaf and twig extracts. Agronomy.

[58] Singleton VL, Rossi JA (1965). Colorimetry of total phenolics with phosphomolybdic–phosphotungstic acid reagents. Am. J. Enol. Vitic..

[59] Chang CC, Yang MH, Wen HM, Chern JC (2002). Estimation of total flavonoid content in propolis by two complementary colorimetric methods. J. Food Drug Anal..

[60] Bakkalbaşı E, Yemiş O, Aslanova D, Artık N (2005). Major flavan-3-ol composition and antioxidant activity of seeds from different grape cultivars grown in Turkey. Eur. Food Res. Technol..

[61] Whitaker JR, Granum PE (1980). An absolute method for protein determination based on difference in absorbance at 235 and 280 nm. Anal. Biochem..

[62] Brand-Williams W, Cuvelier ME, Berset C (1995). Use of a free radical method to evalyate antioxidant activity. LWT Food Sci. Technol..

[63] Sekowski S (2018). Influence of valoneoyl groups on the interactions between Euphorbia tannins and human serum albumin. J. Lumin..

[64] Sekowski S (2017). Interaction of α-synuclein with Rhus typhina tannin—Implication for Parkinson’s disease. Colloids Surf. B Biointerfaces.

